# A practical approach to a low protein diet in Brazil

**DOI:** 10.1186/s12882-016-0305-8

**Published:** 2016-07-29

**Authors:** Denise Mafra, Viviane O. Leal

**Affiliations:** 1 Graduate Program in Medical Sciences and Graduate Program in Cardiovascular Sciences, Federal Fluminense University (UFF), Niterói, RJ Brazil; 2 Graduate Program in Cardiovascular Sciences, Federal Fluminense University (UFF), Niterói, RJ Brazil

**Keywords:** Low protein diet, Chronic kidney disease, Adherence, Brazilians

## Abstract

**Background:**

Chronic kidney disease (CKD) is an emerging health public problem in Brazil. Nutritional counseling with focus on protein restriction is a promising strategy to treatment of nondialysis CKD patients due its effects on slowing renal loss. However, Brazilian people have high protein intake, which is a challenge when low protein diet (LPD) should be prescribed. This review describes a practical approach to the dietetic management of nondialysis CKD patients in Brazil.

**Discussion:**

Although Brazilian cuisine varies greatly by region, Brazil has current trends of incorporating Western dietary habits, including high intake of red meat. Traditional plant-based foods, such as rice and beans, are also important contributors to the high protein content to the Brazilian diet. Thus, a successful implementation of LPD requires adaptation of these dietary habits, with reduction of portion sizes and adequate food substitution options. Intensive nutritional counseling with specialized renal dietitians is also important to improve compliance to the LPD. Moreover, the precarious health system organization and economic problems are barriers to nutritional care, which could be solved with intensive and specialized perspectives of treatment.

**Summary:**

The adherence to protein restriction is important for better metabolic and clinical control of nondialysis CKD patients. Early dietetic attention, nutrition education strategies and intensive specialized nutritional counseling are essential to achieve diet habits that promote adherence to the LPD without excluding cultural characteristics of the Brazilian diet.

## Background

In Brazil, it is estimated that approximately 2.9 million individuals have some degree of renal dysfunction [1]. Therefore, chronic kidney disease (CKD) is a public health problem of great magnitude, and more attention should be paid to it before the patients reach the dialysis stage. Early diagnosis of the disease, in addition to an immediate referral to multidisciplinary groups with an emphasis on medical and nutritional care, can contribute to disease maintenance and have a favorable impact on disease outcomes [2].

Dietary protein restriction is used as a therapeutic measure for nondialysis CKD patients. It was first described 146 years ago and has been used for 60 years. Therefore, a low protein diet (LPD) for these patients is not a new concept. In 1869, Beale [3] proposed that a LPD could ameliorate uremic symptoms. More recently, several studies confirmed the beneficial effects of LPD, such as a reduction of hyperphosphatemia and uremia, amelioration of metabolic acidosis, and protection of the cardiovascular and nutritional status [4–8]. However, the role of dietary protein restriction in slowing CKD progression remains controversial [4]. The results from the few studies on this topic have generated divergent results due, in part, to the variability in adherence to a LPD [9].

Brazilians, in particular, have eating habits that lead to high protein intake and, consequently, difficultly with adhering to the LPD. In a recent study, Souza et al. [10] observed that the food eaten by most adults (>50 %) was white rice, coffee, black beans, refined sugar and French bread. Rice and beans are commonly consumed food in the Brazilian lunch. Moreover, meat is also a food that has significant participation in the Brazilian diet, being the mean meat intake above of 180 g/day [11]. Thus, a hypothetical lunch composed only by rice (120 g), beans (100 g) and beef (90 g) contains approximately 28 g of protein.

In addition to eating habits, another problem is health system organization and economic problems that are barriers to the nutritional care; therefore, improving referral timeliness and regular nutritional monitoring are challenges that nutritionists face. Even with these problems, Brazil has excellent work groups that successfully care for nondialysis CKD patients. Thus, this review discusses several LPD prescription experiences for nondialysis Brazilian CKD patients.

### Low protein diet (LPD)

The protein intake proposed for healthy adults is 0.8 g/kg/day and corresponds to the dietary protein requirements plus a percentage to achieve safe intake [12]. This recommendation is quite similar to the recommendation for the protein intake of nondialysis CKD patients, which is approximately 0.6 [13] to 0.8 g protein/kg/day [4, 13]. However, the term “low protein diet” (LPD) is used to describe this recommendation because a diet restricted to 0.6 g protein/kg/day is 25 % below the recommended level (0.8 g protein/kg/day) [14].

Some diets are more easily ‘adapted’ into LPDs, which renders protein restriction more practical and feasible. Legumes and cereals with a very small amount of dairy products or meats compose the Mediterranean diet. The Okinawa diet contains 30 % green and yellow vegetables, less rice and sugar and more sweet potatoes, small amounts of fish and more soy and legumes. Indeed, the Dietary Approaches to Stop Hypertension (DASH) style diet, with a greater intake of fruits, vegetables and whole grains, may be protective against rapid GFR decline in older white women [15]. In these diets, adaptation to eating habits is adequate and feasible. By contrast, adherence to the LPD may be more difficult in places such as Brazil where the diet is rich in animal protein sources or plant-based foods with considerable amount of protein, such as rice and beans [10, 16].

### LPD in Brazil

Brazil is a developing country with a wide range of cultures and important socio-economic limitations. In general, the eating habits of Brazilians are not desirable and have worsened during the last several decades [17]. Brazil has current trends of incorporating Western dietary habits, which are characterized by a high intake of red meat, sugar, fat and refined grains. However, in an international comparison of dietary intake between Brazilian and American people, Bezerra et al. [18] reported that Brazilian young adults did not consume a diet similar to Americans due to traditional plant-based foods, such as rice and beans, which are important contributors to the Brazilian diet [10].

In a probabilistic sample, Souza et al. [19] observed that the protein intake is relatively high in adults of a metropolitan city of the Rio de Janeiro State (approximately 1.3 g protein/kg/day for males and 1.1 g protein/kg/day for females). In another study developed in a city of the São Paulo State, healthy individuals between 18 and 29 years had a high protein intake (1.6 g/kg for male and 1.4 g/kg female) [20]. Moreover, in a cross-sectional weighted dataset with 2631 Brazilians from the Health Survey for São Paulo, Brazil, Carvalho et al. [21] concluded that red meat processed intake was excessive in almost the entire studied population, and that the consumption of meats, particularly poultry and processed meats, has increased over the last decade to approximately 163 g meat/day. In a recent study, Fisberg et al. [11] observed that the mean meat intake adjusted for sex and age was 182.3 g/day, almost the double of recommended by Brazilian Ministry of Health (100 g/day) [22].

A description of the typical food habits in Brazil is complicated because the country is large and each region has peculiar characteristics. In general, Brazilians have four meals per day: breakfast, lunch, snack and dinner. The typical food for breakfast is coffee, milk, bread with butter or cheese and fresh fruit. For lunch, which is sacred to Brazilians, the typical foods are rice and beans (every day) accompanied by a protein (meat or eggs), vegetables and “farofa”, a toasted flour of manioc. The afternoon snack is coffee or juice accompanied by bread, cookies or typical cakes. Dinner usually consists of the same foods as lunch [23]. The patterns of food acquisition depend on the geographic and socio-economic characteristics, such that the acquisition of dairy, fruits, vegetables and processed meats is associated with a higher household education, whereas the acquisition of manioc, flour, milk and sugar occurs more frequently in less educated households and in rural settings [24].

The national consumption trend of protein-rich foods, particularly meat, is elevated including among families with modest incomes [11]. Therefore, it is easy perceive the challenge of LPD adherence for nondialysis Brazilian CKD patients.

### LPD for nondialysis CKD patients: a Brazilian experience

#### Referring patients

Nephrologists usually forward nondialysis CKD outpatients together with their detailed information about GFR (estimated GFR or creatinine clearance by 24 h-urinary output) and albuminuria (determined by 24 h-urinary output more frequently than the urinary spot). In 2013, the number of nephrologists was estimated in 2,885 (1.08 % of doctors in Brazil) [25]. More than 70 % of nephrologists have obtained the specialist title after conclusion of a Medical Residency Program. The majority of Brazilian nephrologists acts in the Public sector, being the main focus of their work the outpatient care in medical offices associated with other activities (64.9 %), mainly in dialysis units [26]. In Brazil, dialysis is offered by private clinics accredited to “Sistema Único de Saúde”, a health care model with free and full coverage to every citizen, and the government reimburses these clinics for each dialysis session made.

In the specific case, we are a group of four dietitians (students or graduated in Master or PhD programs) supervised by a dietitian PhD and expert in the Renal Nutrition. The activities are developed in a Nutrition Faculty in Federal University Fluminense, a public University. Thus, we can follow CKD patients followed by Public and Private Services through presentation of GFR and albuminuria results. Our group considers the presentation of these biochemical measurements mandatory before starting nutritional counseling.

All patients who come to us are nutritionally followed; however, due to precarious organization of Brazilian health system there are no estimates about the number of patients followed by dietitians among all those with CKD (in locally, regionally or nationally level). Moreover, it is not possible to ensure the reference of these patients to other professionals (endocrinologists, physiotherapists, physical educators and psychologists, for example).

### Patients and LPD prescription

All referred patients, except palliative cancer patients with a short life expectancy (<6 months, estimated by oncologist), are included in the dietary LPD program. Only nutritionists are responsible for nutritional counseling and LPD prescribing (in Brazil is defined by Law that diet prescription is an activity that can only be performed by dietitians).

### Outpatient organization

During the first nutritional appointment, the following information is obtained:

1. A survey of relevant demographic, clinic and socio-economic data, such as age, comorbities, professional activity, per capita income, number of people living in the house, and number of home-cooked meals. For biochemical parameters in addition to GFR and albuminuria, the measurements of glycemic control and lipid profile are useful for determining which foods are better options to increase dietary energy intake, if necessary.
2. Evaluation of the nutritional status through measures of weight and stature for body mass index calculation, arm circumference and skinfolds (or percentage of body fat) and muscle arm circumference estimation and waist circumference for cardiovascular risk evaluation.
3. Assessment of food intake by 24-h food recall. Measuring tools and photographic albums are used to improve the accuracy of the patient’s record. We show tubes containing different amounts of salt and food replicas to help the patient’s memory.
4. Explanation about the goals of nutritional counseling during the nondialysis CKD stage with a focus on protein intake. During this time, educational material that was specifically developed for nondialysis CKD patients is provided. This educational material (“hornbook”) also contains dietary guidelines to control salt intake, such as avoiding processed spices, restriction of salt and preference for natural seasonings, and hyperkalemia, such as a preference for fresh fruits with lower potassium content and double-cooking for vegetables. The first appointment is very long, approximately 60–90 min. Therefore, the patients are asked to make an appointment for the next week to receive the personalized nutritional plan.

During the second nutritional appointment, which is held one week later, the following occur:

1. Answer possible questions about treatment goals, such as “How long will I need to do this diet?” or “With this diet, my kidney will function normally again?” or “When and how much can I eat fruits with more potassium?”
2. Deliver and explain the personalized nutritional plan, such as portions, replacement list, and cooking methods.
3. Adjust the appointment interval to 1, 2 or 3 months, depending on the patient’s needs.

During the third nutritional appointment, the following occur:

1. Evaluation of weight and anthropometric changes.
2. Evaluation of LPD adherence through 24-h food recall and biochemical measurements, such as serum creatinine and urea, GFR and proteinuria. Every six months, a measurement of protein nitrogen appearance is requested; however, many economic problems can delay this requirement.
3. Adjustment of dietary prescription, if necessary. If adherence is considered “good” (protein intake between 90 and 110 % of dietary prescription), nutritional appointments are made for every three months. If adherence is <90 % or >110 %, nutritional appointments occur for each month until the desirable LPD adherence is reached.

#### Key point

According to the NKF [13], regular monitoring at 1- to 3-month intervals (4 visits per year) should be a routine component of nutritional care.

In the subsequent appointments, the following occurs:

1. At each visit, a complete clinical, anthropometric and dietetic evaluation is performed and the dietary plan is adjusted, if necessary. A simplified scheme with routine nutritional care is described in Table 1.

**Table 1:** Routine of nutritional care

Forwarding Order: nephrologists to dietitians Biochemical mandatory measurements: GFR and albuminuria Biochemical additional measurements: lipid and glycemic profile
Patients Target: all referred patients, except palliative cancer patients (<6 months of life expectance)
Diet Type: adapted to Brazilian eating habits and patient´s food preferences Energy intake: approximately 30 to 35 kcal/kg/day Protein intake: approximately 0.6 to 0.8 g/kg/day Nutrient calculation: own software with foods arranged in standard servings per food groups Replacement list of food: available Estimation of portion sizes by measuring tools and photograph albums Recipes with low protein content
Outpatient organization First nutritional appointment * survey of demographic, clinical, socio-economic and biochemical data * nutritional status evaluation (BMI, %BF, MAC, WC) * assessment of food intake (24-h food recall) * explanation of the goals of nutritional counseling and delivery of a book with dietary guidelines about protein, potassium and salt intake Second nutritional appointment (one week later) * Personalized nutritional plan Subsequent nutritional appointments (one, two or three months apart) * nutritional status evaluation * LPD adherence evaluation (24-h food recall, biochemical measurements and PNA) * Adjustments of dietary prescriptions, if necessary

*GFR* glomerular filtration rate, *BMI* body mass index, *%BF* percentage of body fat, *MAC* muscle arm circumference, *WC* waist circumference, *LPD* low protein diet, *PNA* protein nitrogen appearance

### Types of diets prescribed

#### Energy

A diet containing 35 kcal/kg/day for patients < 60 years old and 30 kcal/kg/day for patients >60 years old is prescribed [13]. For overweight and obese patients, 30 kcal/kg/day may be considered. For patients who need weight repletion, energy intake > 35 kcal/kg/day should be implemented.

#### Protein

A diet containing 0.6 g protein/kg/day (>50 % of high biologic value) is prescribed [13]. For catabolic patients, such as patients with involuntary weight loss, diabetics with glycemic uncontrolled and those with protein-energy wasting, a diet containing up to 0.8 g protein/kg/day may be prescribed [4, 13]. The very-low protein diets are not implemented because the Brazilian Public Health System does not provide free ketoanalogs distribution.

The weight considered for prescription of energy and protein is the actual or desirable/ideal weight. Desirable/ideal weight is used in cases of obesity or protein-energy wasting and it corresponds to the weight that should be achieved considering a BMI value within the normal range (ideal/desirable weight = BMI desired X height (m^2^)) [27]. This is a specific recommendation of the Brazilian nutritional guideline for treating of nondialysis CKD patients [27], which use the American guidelines as reference [13].

#### Key points

The correct implementation of LPD, which includes careful attention to the amount of protein of high biological value, proper energy intake and other nutrients, such as vitamins and micronutrients, does not induce PEW [14, 15, 28]. Adequate energy intake during protein diet restriction may be difficult. Therefore, the use of nonprotein calorie supplements [29] or strategies for increasing protein-free foods are important and should account for any associated patient comorbities, such as increased intake of olive oils and butter for diabetic patients with PEW or increasing the use of honey and manioc flour for nondiabetic patients with PEW.

An example of a personalized nutritional plan for a nondiabetic patient and its possible variations is presented in Table 2. For diabetic patients, national guideline [30] recommends that up to 10 % of the total energy intake may be from sucrose (and in this diet example, this percentage is approximately 12.1 %). Thus, portions of sugar and/or flour manioc could be reduced and other foods, such as fruits or vegetables with lower glycemic index, can be included to achieve the total energy value (1800 kcal), according to TACO [31].

**Table 2:** Nutritional plan example

Meals		Foods	Energy (Kcal)	Protein (g)
Breakfast				
50 g 8 g 100 ml 12 g 100 g	Bread (one portion) Butter (two teaspoon) Coffee (half cup) Sugar (two teaspoon) Fruit (one portion) Example:1 “French” bread with butter, 1 cup (200 ml) coffee with sugar + half papaya		125 60 0 48 58	4 0 0 0 0.8
Lunch				
25 g 75 g 15 ml 90 g 40 g 20 g 50 g 100 g 10 ml	Green salad (free) Braised mix vegetables (three tablespoon) Olive oil (three teaspoon) Rice (three tablespoon) Beans (half scoop) Flour manioc (1 tablespoon) Protein (half portion) Fruit (one portion) Oil for cooking Example: Green salad + braised pumpkin and carrot + rice + beans + flour manioc + roasted chicken thigh + one banana		5.5 22.5 135 125 31 71 90 58 90	0.4 1.0 0 1.8 2.0 0.3 10.5 0.8 0
Snack				
200 ml 12 g 30 g 8 g 100 g	Fruit refreshment (one cup) Sugar (two teaspoon) Cookie (one portion) Butter (two teaspoon) Fruit (one portion) Example: One cup of lemonade + five cookies + one apple		25 48 127 60 58	0 0 2.8 0 0.8
Dinner				
25 g 75 g 15 ml 90 g 20 g 50 g 100 g 10 ml	Green salad (free) Braised mix vegetables (four tablespoon) Olive oil (three teaspoon) Rice (three tablespoon) Flour manioc (1 tablespoon) Protein (half portion) Fruit (one portion) Oil for cooking Example: One watercress and spinach saucer salad + braised broccoli, cauliflower + beet + manioc (one piece, substitution for flour) + rice + one small steak + one orange		5.5 22.5 135 125 71 90 58 90	0.4 1.0 0 1.8 0.3 10.5 0.8 0
	Sum all meals		1834	40
	Values adjusted to body weight		30.5	0.66

Male, 61 years old, 60 kg, 1.6 m (BMI: 23.4 kg/m^2^), with hypertension as etiology of CKD. GFR: 48 mL/min/1.73, Albuminuria: 130 mg/day and normal K and P plasma levels

Normal values of body fat and arm circumference and increased (96 cm) waist circumference

Personalized plan: Energy: 30 × 60 kg = 1800 kcal/day; Protein: 0.6 × 60 g = 36 g/day

Calories and protein according to ref 33

### Improving LPD adherence

Some strategies are designed to improve LPD adherence, such as the following:

1. Dietary plan is elaborated based on habitual meals using data obtained from the 24-h dietary recall and other information about habitually consumed foods and a schedule of meals. For example, if soy protein intake is not a habit, the diet will include fish, eggs, meat or chicken protein. If a patient does not eat dinner, options for snacking in the evening hours are provided. Well-recognized dietary patterns, such as DASH or the Mediterranean diet, could be a strategy; however, they are not consistent with Brazilian eating habits. Therefore, we prefer to make adaptations to the usual dietary intake of nondialysis CKD patients.
2. A replacement list for each food group is provided to avoid food monotony. For example, small portions of protein include a small piece of steak, three meatballs, one egg, or one small chicken leg.
3. Measuring tools (replicas of food and kitchen utensils) and photographic albums are used to estimate portion sizes.
4. With follow-up, patients are stimulated to improve their meals with recipes that are specially designed for nondialysis CKD stage [32].

### Unrestricted meals

Unrestricted meals are not allowed. On special occasions, such as parties and Christmas, better options among the meals served are indicated, such as fruits with a lower potassium content during Christmas and cakes with fruit fillings during birthdays.

### Final remarks

Despite the controversy regarding the effectiveness of LPD for slowing CKD progression, the adherence to protein restriction is important for better metabolic and clinical control. Therefore, motivating patients to adhere to dietary protein restriction is an essential point for the management of nondialysis CKD patients [14]. To maintain the LPD over time, the diet prescribed must be pleasant, varied and not too restrictive [14, 33]; therefore, intensive counseling by a skilled dietitian is necessary.

## Abbreviations

CKD, chronic kidney disease; GFR, glomerular filtration rate; LPD, low protein diet; PEW, protein-energy wasting
